# Editorial: Special Issue “Genetic Newborn Screening”

**DOI:** 10.3390/genes16091006

**Published:** 2025-08-26

**Authors:** Hisahide Nishio, Hiroyuki Awano

**Affiliations:** 1 Faculty of Rehabilitation, Kobe Gakuin University, Kobe 651-2180, Japan; 2 Organization for Research Initiative and Promotion, Tottori University, 86 Nishi-cho, Yonago 683-8503, Japan; awano@tottori-u.ac.jp

## 1. Introduction: Short History of Newborn Screening (NBS)

Traditional newborn screening (NBS) is performed by measuring disease-specific biomarkers using the latest state-of-the-art technologies. This type of NBS is referred to as biomarker NBS.

In the mid-20th century, NBS for phenylketonuria (PKU) was first introduced as a public health program in the United States. PKU is a genetic disorder that causes severe intellectual disability, with a specific biochemical signature: hyperphenylalaninemia. In 1963, Robert Guthrie developed a simple bacterial inhibition screening assay to detect PKU patients with hyperphenylalaninemia using dried blood spots (DBS) on filter paper [1,2]. PKU patients diagnosed through screening are treated with a low-phenylalanine diet.

NBS tests for hypothyroidism, adrenal hyperplasia, and cystic fibrosis using radioimmunoassays were also subsequently developed, leading to early treatment of these diseases/disorders [3,4,5,6]. Radioimmunoassays consisting of an endogenous substance mixed with an exogenous radioisotope-labeled substance are used to measure endogenous substance levels with reference to the isotope level in an immune complex.

Up to the 1990s, disease-specific diagnostic tests were limited by the fact that they provided information on only one disease per test. However, the advent of electrospray tandem mass spectrometry with automated sample injection has enabled high-throughput screening for the measurement of characteristic metabolites (amino acids and acyl-carnitines) of various congenital metabolic disorders [7].

In recent years, NBS has been initiated to detect abnormalities at the genomic DNA level in patients with spinal muscular atrophy (SMA) and severe combined immunodeficiency (SCID) [8]. This new type of NBS is referred to as genetic NBS and is generally performed by means of polymerase chain reaction (PCR). Genetic NBS does not rely on disease-specific biomarkers, and the same analytical method can therefore be used to identify patients with any genetic disorder. These medical advances in genetic NBS will contribute to the healthy development of children suffering from a wide range of genetic disorders, in addition to children with SMA.

## 2. Research Published in the Special Issue “Genetic Newborn Screening”

In this Special Issue, new methodologies for genetic NBS systems, the new target diseases of genetic NBS programs, the molecular pathological mechanisms newly identified through genetic NBS research, and accurate epidemiological findings based on genetic NBS data are presented. The information introduced in the following section highlights some key points from each study.

### 2.1. NBS for SMA

#### 2.1.1. Implementation of an NBS Program for SMA

SMA (more precisely, 5q-SMA) is a common and severe neuromuscular disease caused by the homozygous deletion of the survival motor neuron 1 (*SMN1*) gene [9]. SMA is inherited in an autosomal recessive manner [9]. The incidence of SMA is approximately 1 in 10,000 to 20,000 live births, with the carrier frequency being 1/40 to 1/70 in the general population. If treatment is initiated early, newly developed drugs may improve motor function in infants with SMA. To enable early treatment, many countries have initiated NBS programs for SMA [9].

In one article in our Special Issue, entitled “Integrated Approaches and Practical Recommendations in Patient Care Identified with 5q Spinal Muscular Atrophy through Newborn Screening” by Romanelli Tavares et al., the authors report on how they implemented an NBS program in Brazil [10]. The findings of this report will be of particular utility for countries planning to implement NBS programs for SMA.

#### 2.1.2. Follow-Up of SMA Patients Identified Through NBS

*SMN2*, a paralog of *SMN1*, modifies the phenotype of SMA to some degree. For example, the increased copy number of *SMN2* may reduce the severity of SMA. Measuring the copy number of *SMN2* is thus of great utility in predicting the clinical course of patients with SMA [9]. The efficacy of NBS in enabling early treatment is reflected in better clinical outcomes than those predicted based on *SMN2* copy number.

In the article entitled “Newborn Screening for Spinal Muscular Atrophy: A 2.5-Year Experience in Hyogo Prefecture, Japan”, Sonehara et al. report on the follow-up of SMA patients identified through NBS [11]. They identified three infants with SMA with an *SMN1* deletion using NBS. Case 1 possessed two copies of *SMN2* and presented with SMA-related symptoms (hypotonia and respiratory distress) at diagnosis. Case 2 also possessed two copies of *SMN2* but presented with no SMA-related symptoms (asymptomatic patient), while Case 3 possessed four copies of *SMN2* exon 7 and presented with no SMA-related symptoms. All of these patients were treated with newly developed drugs, leading to improvements in their motor and respiratory functions. Additionally, Sonehara et al. compared the clinical outcomes between patients with SMA identified through NBS and patients identified based on SMA-related symptoms and found that the latter did not benefit from early diagnosis and early treatment. The authors concluded that NBS enabled the early diagnosis and treatment of SMA, leading to better outcomes.

#### 2.1.3. Homozygous *SMN2* Deletion in the Japanese Population

As noted in Section 2.1.2, the *SMN2* gene is a recognized modifier of SMA. However, our knowledge regarding the function of *SMN2*—other than its modification of SMA phenotypes—remains limited. Discussions regarding the relationship between homozygous *SMN2* deletion and motor neuron diseases, including amyotrophic lateral sclerosis, have primarily been based on retrospective epidemiological studies of these diseases, and the precise relationship remains inconclusive. To date, however, no studies have investigated its incidence in the general population of Japan.

In the article entitled “Real-Time PCR-Based Screening for Homozygous *SMN2* Deletion Using Residual Dried Blood Spots”, Bouike et al. report that the incidence of homozygous *SMN2* deletion in Japan is approximately 1 in 20 individuals (5%) [12]. This incidence rate is much higher than that of homozygous *SMN1* deletion and may reflect the vulnerability of the *SMN2* region.

### 2.2. Severe Combined Immunodeficiency (SCID) and Sickle Cell Disease (SCD)

#### 2.2.1. T Cell Receptor Excision Circles (TRECs) and Kappa-Deleting Recombination Excision Circles (KRECs) in SCID

SCID is one of the most severe forms of primary immunodeficiency and is characterized by the absence of functional T lymphocytes. It is life-threatening when diagnosed in its later stages. Affected infants die because of overwhelming bacterial, fungal, or viral infections in infancy [13]. Regarding NBS for SCID, the copy number of TRECs has been used as a surrogate marker of thymic function and lymphocyte maturation [14]. NBS programs for SCID in the USA using a TREC assay indicated an incidence of 1 in 100,000 births [15].

In the article entitled “A Unique Comprehensive Model to Screen Newborns for Severe Combined Immunodeficiency—An Ontario Single-Centre Experience Spanning 2013–2023”, Al Ghamdi et al. report that from August 2013 to April 2023 in their center’s densely populated catchment area in Canada, 162 newborns with low TREC levels were identified, including 10 cases with SCID [16]. Follow-up revealed other causes of low TREC levels, including non-SCID T cell lymphopenia (secondary/reversible or idiopathic causes and syndromic conditions) and prematurity. They demonstrated that NBS-positive SCID cases included pathological conditions other than SCID.

The copy number of TRECs is a marker for T cell lineage development; in comparison, the copy number of KRECs is a marker for B cell lineage development [17]. Kimizu et al. reported their experience of NBS for SCID and SMA in an article entitled “Multiplex Real-Time PCR-Based Newborn Screening for Severe Primary Immunodeficiency and Spinal Muscular Atrophy in Osaka, Japan: Our Results after 3 Years” [18]. Their NBS programs for SCID targeted the copy numbers of TRECs and KRECs. The authors concluded that simultaneously assessing the levels of TRECs and KRECs presents significant advantages when diagnosing various immunodeficiencies.

#### 2.2.2. Modularity of Genetic NBS Technologies with Multiplex Real-Time PCR

As described above, the NBS system of Kimizu et al. used multiplex real-time PCR, enabling screening of multiple pathological conditions (or diseases) [18]. When using real-time PCR, different fluorescent dyes can be detected simultaneously, and by allocating different fluorescent dyes to different genes, real-time PCR can assay different genes. Kimizu et al. succeeded in measuring the gene copy numbers of TRECs, KRECs, and *SMN1* simultaneously.

In 2018, a large-scale pilot genetic screening program for SMA and cystinosis was initiated in Germany using a 384-well qPCR-based high-throughput screening system [19,20]. However, 2 years later, cystinosis screening was discontinued because of the condition’s low incidence, approximately 1:100,000 newborn babies [21,22]. Cystinosis screening was then replaced by the TREC test for SCID, with the beta-globin (HBB) test for SCD added thereafter.

In the article entitled “A Modular Genetic Approach to Newborn Screening from Spinal Muscular Atrophy to Sickle Cell Disease—Results from Six Years of Genetic Newborn Screening”, Bzdok et al. demonstrated that a target disease in NBS can be easily replaced with another disease using multiplex real-time PCR that detects multiple fluorescent dyes simultaneously [22]. The modularity of multiplex real-time PCR enables the simple adaptation of new genetic assays using existing laboratory equipment.

#### 2.2.3. Melting Curve Analysis for the Detection of Mutant Hemoglobin S (HbS) in SCD

SCD is a common hemoglobin disorder caused by the presence of a mutant hemoglobin (HbS) resulting from a valine to glutamic acid substitution at the sixth amino acid position of HBB [23]. SCD is inherited in an autosomal recessive manner but is characterized by a variety of manifestations ranging from acute generalized pain to early-onset stroke, leg ulcers, and the risk of premature death from multi-organ failure. The gene frequency of HbS is highest in West African countries, with 1 in 3–4 individuals (25–30%) being carriers of HbS; its frequency in European populations is variable, however [23]. The prevalence of SCD in developed countries is increasing partly because of migration from countries characterized by high prevalence.

Bzdok et al. reported the use of melting curve analysis using a real-time PCR system to detect the variant alleles of HBB [22]. They used melting curve analysis as a primary screening test (first-tier screening test) and high-performance liquid chromatography (HPLC) as the second-tier test. Screening for SCD is commonly performed using HPLC or matrix-assisted laser desorption/ionization time-of-flight mass spectrometry; however, genetic testing (herein, melting curve analysis) combined with HPLC confirmation may be more cost-effective and time-saving for high-throughput screening compared with the previously mentioned methods.

### 2.3. NBS for Peroxisomal Disorders

#### 2.3.1. X-Linked Adrenoleukodystrophy (X-ALD)

X-ALD is the most common peroxisomal disorder, with an estimated birth prevalence (or incidence) of ~1 in 20,000 [24]. X-ALD is caused by mutations in the ATP-binding cassette subfamily D member 1 (*ABCD1*) gene, located on the X chromosome, which produces the adrenoleukodystrophy protein (ALDP). X-ALD increases the concentrations of very long-chain fatty acids (VLCFAs) in the plasma, in addition to adrenal and nervous tissues [24]. Neurological manifestations vary widely, even within the same family, ranging from a rapidly progressive childhood cerebral form to a more slowly progressive adrenomyeloneuropathy in adults [24].

A three-tiered NBS program for X-ALD was initiated in California, USA [25]. The first-tier NBS involved the analysis of C26:0-lysophosphatidylcholine (C26:0-LPC) in DBS samples using flow injection analysis tandem mass spectrometry. The second-tier NBS involved the use of liquid chromatography–tandem mass spectrometry to provide a more precise measurement of C26:0-LPC in DBS samples. For the third-tier NBS, all second-tier positive DBS samples were subjected to nucleotide sequencing analysis of the *ABCD1* gene. The ALD birth prevalence (or incidence) in California, USA, was 1 in 14,397 males [25].

#### 2.3.2. Zellweger Spectrum Disorder (ZSD)

The implementation of NBS for X-ALD has not only enabled the early identification of male patients with X-ALD but also facilitated the identification of patients with other genetic metabolic diseases beyond X-ALD that also induce an increase in C26:0-LPC, including ZSD [25].

In the article entitled “Newborn Screening for X-Linked Adrenoleukodystrophy (X-ALD): Biochemical, Molecular, and Clinical Characteristics of Other Genetic Conditions”, Mares Beltran et al. focused on inherited metabolic diseases other than X-ALD identified through NBS for this condition [26]. Their nine cases demonstrated significantly elevated C26:0-LPC levels, highlighting its utility as a biomarker for peroxisomal disorders. Based on their genetic analysis, seven out of nine cases were proven to be patients with ZSD.

ZSD is an autosomal recessive disorder caused by various defects in any of the 13 *PEX* genes [26], which encode proteins involved in peroxisome biogenesis and proliferation. Defects in these genes impair normal peroxisome production and maintenance, affecting multiple metabolic pathways, including the catabolism of VLCFAs, branched-chain fatty acids, plasmalogen synthesis, and bile acid synthesis, including cholic acid.

Patients with ZSD present with a wide range of clinical symptoms from mild to severe [26], including poor feeding, developmental delay, growth retardation, hearing loss, and visual impairment. Other physical findings include frontal bossing and hepatomegaly. ZSD presents a diagnostic challenge because of its heterogeneous clinical manifestations.

Beltran et al. concluded that the identification of children with ZSD and atypical patterns of *ABCD1* variants is a secondary benefit of NBS for X-ALD, leading to earlier diagnosis, prompt therapeutic initiation, and more accurate genetic counseling [26].

### 2.4. Alternative Specimens for Genetic Screening: Oral Swabs

In the article entitled “Establishing a Standardized DNA Extraction Method Using NaCl from Oral Mucosa Cells for Its Application in Imprinting Diseases Such as Prader–Willi and Angelman Syndromes: A Preliminary Investigation”, Letícia Fonseca et al. demonstrated that the simple NaCl extraction method is suitable for extracting DNA from a buccal swab sample [27]. Sample collection and genomic DNA extraction from buccal swabs are non-invasive techniques and may represent reliable alternatives to invasive and uncomfortable blood collection for subjects and sample collectors. The authors applied this DNA extraction method to new diagnostic technologies for Prader–Willi syndrome (PWS) and Angelman syndrome (AS), with the method combining DNA extraction from buccal swabs and methylation-sensitive high-resolution melting (MS–HRM) analysis. The MS–HRM was performed with bisulfite-treated DNA, leading to C-to-U converted DNA.

PWS and AS are epigenetic diseases caused by a loss of imprinting (LOI) [28]. The pathological manifestations of these diseases are caused by an LOI located in the imprinting center of the 15q chromosome. PWS and AS affect approximately 1 in 15,000 newborns. Both diseases are associated with developmental, behavioral, and psychiatric problems [29].

Since the introduction of NBS by Guthrie [1], it has been implemented according to the following principles: (1) using dried blood samples from infants, soaked into disks of filter paper (DBS), and (2) detecting diseases for which effective early treatment is available. The NBS program reported by Letícia Fonseca et al. differs from such conventional NBS programs in that it replaces infant samples with buccal swab samples and applies NBS to diseases for which there is no cure; however, treatments for these diseases are likely to become available in the near future, and NBS techniques may then prove effective in detecting patients with these diseases.

## 3. Conclusions

This Special Issue focuses on studies of the technological development of NBS. Each article is the result of the ingenuity of the researchers. However, considering the fact that NBS is a public health program that enables early treatment, it is essential to form a social consensus on its economic feasibility and to raise social awareness of the disease to be screened. Regarding SMA, discussions have begun on the economic feasibility of NBS [30] and the social awareness of the disease [31].

Another important issue is the need to raise awareness of the limitations of NBS. False-positive and false-negative NBS results should be acknowledged as limitations of the assay systems. It is also necessary to make it clear that NBS can only be used to detect a limited number of diseases; e.g., the term “NBS for SMA” is widely used, but “SMA” herein refers to “5q-SMA”, and “NBS for SMA” should thus be replaced by “NBS for 5q-SMA”.

Few families know of the existence of SMA other than 5q-SMA (non-5q-SMA) or recognize that the current NBS for SMA is for 5q-SMA alone [32]. The parents of babies with non-5q-SMA might face much greater challenges compared with the parents of those with 5q-SMA. These parents may believe that SMA has been ruled out because the widely implemented NBS for SMA (more precisely, 5q-SMA) was negative, and a subsequent diagnosis of non-5q-SMA, which cannot be cured with currently available drugs, would be devastating for them.

In conclusion, the articles included in this Special Issue demonstrate the progress of technological development and program implementation in NBS; it is also important, however, to recognize that NBS presents challenges other than technical or implementation issues, including awareness of target diseases.

## Data Availability

Not applicable.
